# City-level greenness exposure is associated with COVID-19 incidence in China

**DOI:** 10.1016/j.envres.2022.112871

**Published:** 2022-06

**Authors:** Wenjia Peng, Yilin Dong, Meihui Tian, Jiacan Yuan, Haidong Kan, Xianjie Jia, Weibing Wang

**Affiliations:** a School of Public Health, Fudan University, Shanghai, China; b Key Laboratory of Public Health Safety (Ministry of Education), Fudan University, Shanghai, China; c School of Public Health, Bengbu Medical College, Bengbu, Anhui, China; d IRDR-ICoE on Risk Interconnectivity and Governance on Weather/Climate Extremes Impact and Public Health, Fudan University, Shanghai, China

**Keywords:** Greenness, NDVI, COVID-19, Incidence, China

## Abstract

Accumulating studies have suggested an important role of environmental factors (e.g. air pollutants) on the occurrence and development of coronavirus disease 2019 (COVID-19). Evidence concerning the relationship of greenness on COVID-19 is still limited. This study aimed to assess the association between greenness and COVID-19 incidence in 266 Chinese cities. A total of 12,377 confirmed COVID-19 cases were identified through February 29th, 2020. We used the average normalized difference vegetation index (NDVI) during January and February 2020 from MOD13A2 product, to represent the city-level greenness exposure. A generalized linear mixed-effects model was used to estimate the association between NDVI exposure and COVID-19 incidence using COVID-19 cases as the outcome. We evaluated whether the association was modified by population density, GDP per capita, and urbanization rate, and was mediated by air pollutants. We also performed a series of sensitivity analyses to discuss the robustness of our results. Per 0.1 unit increment in NDVI was negatively associated with COVID-19 incidence (IRR: 0.921, 95% CI: 0.898, 0.944) after adjustment for confounders. Associations with COVID-19 incidence were stronger in cities with lower population density, lower GDP per capita, and lower urbanization rate. We failed to detect any mediation effect of air pollutants on the association between NDVI and COVID-19 incidence. Sensitivity analyses also indicated consistent estimates. In conclusion, our study suggested a beneficial association between city-level greenness and COVID-19 incidence. We could not establish which mechanisms may explain this relationship.

## Highlights

• The study is the first to discuss the association of NDVI with COVID-19 incidence in 266 Chinese cities.
• NDVI exposure was negatively associated with COVID-19 incidence.
• Stronger protective effect was observed in cities with lower population density.
• Stronger protective effect was observed in cities with lower GDP per capita.
• Stronger protective effect was observed in cities with lower urbanization rate.

## 1 Introduction

Following the outbreak of SARS-CoV in 2002 and the MERS-CoV in 2012, another highly pathogenic zoonotic pathogens, named coronavirus disease 2019 (COVID-19), was found to be epidemiological in contact with the human seafood market in Wuhan, China in late December 2019 (Lu et al., 2020). On March 11, 2020, COVID-19 had officially been declared as an epidemic public health emergency of international concern by the World Health Organization (Mahase, 2020). Within one month, the COVID-19 virus spread rapidly throughout China during the period of the Chinese New Year (Adhikari et al., 2020). Epidemics in China arrived at the peak in late January and early February 2020. Since then, it has increased so exponentially that developed into a global pandemic and has affected huge numbers of people all over the world (Callaway et al., 2020). After several strict major implementations including keeping social distance, wide-reaching mobility restrictions, and gatherings canceling, China successfully controlled the spread of the epidemic. By the middle of March, the total number of confirmed cases inside China declined drastically and the epicenter of the virus has now shifted to Europe (Fisher and Wilder-Smith, 2020). As of September 20, 2021, a total of 229, 329, 042 confirmed cases and 4,705,890 deaths were detected in 223 countries, with a total of 95,689 cases and 4,636 deaths in China. The global pandemic of COVID-19 has led to a negative effect on human health, economic development, and social stability (Meng et al., 2021). Identifying modifiable factors for preventing the spread of COVID-19 is particularly important.

Environmental factors play a large role in the occurrence and development of a variety of health outcomes. Built environment refers to the artificially constructed structures and infrastructure to provide for human activities, including land use, transport network, and green space. Green space is defined as open pieces of land which are covered by trees, grasslands, or other vegetation (de Keijzer et al., 2019b). In recent years, an accumulating body of evidence suggested that the presence of green space is beneficial for a broad range of health outcomes including diabetes (Yang et al., 2019b), metabolic syndrome (de Keijzer et al., 2019a), hypertension (Yang et al., 2019a), obesity (Huang et al., 2020; Sarkar, 2017), cardiovascular disease (Jia et al., 2018), depression (Gascon et al., 2018; Pun et al., 2018) and mortality (James et al., 2016; Kasdagli et al., 2021). However, previous studies mainly focused on chronic non-infectious diseases and mental disorders. Evidence concerning the relationship of greenness with infectious disease is still limited. Although, the underlying mechanisms by which greenness benefits health are not fully elucidated, several pathways have been investigated. Firstly, access to more green could reduce certain harms, such as exposure to air pollution and heat (Gunawardena et al., 2017; Hankey and Marshall, 2017). Secondly, living in greener areas might promote individuals to participate in physical activity (James et al., 2017; Sadeh et al., 2019), which is critical to help the immune system against virus (Shahrbanian et al., 2020). Thirdly, exposure to more greenness is beneficial for relieving mental and physiologic stress (Markevych et al., 2017).

To the best of our current knowledge, there has been no study to examine the association between greenness exposure and COVID-19 incidence in China. Therefore, we utilized an ecological study based on 266 Chinese cities to explore: 1) whether greenness exposure was negatively associated with COVID-19 incidence; 2) whether the association was modified by potential covariates; 3) whether the association was mediated by air pollutants.

## 2 Methods

All data in the study were obtained from publicly available sources. Table S1 presented the descriptive and source of the outcome, exposure, potential covariates, air pollutants, and meteorological factors.

### 2.1 COVID-19 cases

As of February 29th, 2020, the total number of COVID-19 confirmed cases in China was 79,824, with 66,907 cases in Hubei province, accounting for more than 80% of total cases. Therefore, cities in Hubei province were not included to avoid bias. City-level COVID-19 confirmed cases were collected from the National Health Commission and the Provincial Health Commissions from January 1st to February 29th, 2020, corresponding to the first wave of COVID-19 infections in China, including 266 Chinese cities from 20 provinces, 4 autonomous regions, and 4 municipalities directly under the Central Government.

### 2.2 Exposure measurements

We quantitatively assessed the city-level greenness using normalized difference vegetation index (NDVI) from vegetation product of Moderate-resolution imaging spectroradiometer (MODIS) sensor aboard the National Aeronautics and Space Administration (NASA) Terra satellite, namely MOD13A2, available at https://ladsweb.modaps.eosdis.nasa.gov/search/. MOD13A2 product provides vegetation index at 1 km spatial resolution. The algorithm for this product was based on the best available pixel value from all the acquisitions. NDVI values ranged from −0.2 to 1, with higher values indicating more greenness. We downloaded NDVI data from January 1st to February 29th, 2020. The mean value of NDVI was assigned to each city, representing the greenness exposure. The extraction of vegetation index was done using R statistical software, Version 4.0.3 (University of Auckland, New Zealand). The negative value was set to NA (not available) which represented water features.

### 2.3 Potential covariates

According to prior knowledge, we collected city-level variables from numerous public sources to adjust for confounding bias. From the 7th national population census, we obtained the following city-level covariates: total population, the proportion of older adults aged over 65 years old (older people), male to female ratio (gender ratio), average education years of the population aged over 15 years old (education years), urbanization rate. Population density (person/sq. km.) was calculated using total population divided by land area. From the China city statistical yearbook of 2019, we collected GDP per capita, hospital beds, and doctors per 1,000 people (doctors).

A government response index (GRI) indicator from publicly-accessible data was collected by the Coronavirus Government Response Tracker at

https://www.bsg.ox.ac.uk/research/research-projects/covid-19-government-response-tracker. The GRI was calculated using a series of indicators about governments’ policy response to the COVID-19 pandemic, mainly including containment and closure, economic response, and health systems from January 1st, 2020. The GRI ranges from 0 to 100, with higher values indicating a higher overall response level. We collected the average provincial level GRI within the time span of 60 days from January 1st to February 29th, 2020.

We also considered the influence of population movement on COVID-19 incidence. An intra-city movement intensity, defined as the proportion of people traveling within cities, was derived from Baidu map migration big data (http://qianxi.baidu.com/) from January 1st to February 29th, 2020. Population movements are anonymously collected at the city level based on individual geographic location.

### 2.4 Air pollutants and meteorological data

We collected air pollutants from the Bureau of Ecological Environment of each city, including fine particulate matter (PM_2.5_), nitrogen dioxide (NO_2_), and carbon monoxide (CO). The daily concentrations of PM_2.5_, NO_2_, and CO were available from January 1st to February 29th, 2020. We averaged daily concentrations of these pollutants and then aggregated them into each city. Meteorological data on daily mean temperature (°C) and daily mean relative humidity (%) during the same period were also collected from the China Meteorological Data Sharing Service System.

### 2.5 Statistical analysis

A generalized linear mixed-effects model was used to evaluate the association of NDVI and COVID-19 incidence with a random intercept for provinces to account for a potential correlation in cities within the same province. We included a log population offset in the model. Hence, the model could be expressed as follows:

$$Log\left(E\left(incident\;cases\right)\right)=\beta_{0}+\overset{p}{\underset{j=1}{\sum }}\beta_{j}\cdot x_{j}+\;random\;intercept\left(province\right)\;+offset\left(log\left(population\right)\right)$$

where E (⋅) is an expected value of incident cases, $\beta_{0}$ is the intercept, $\beta_{j}$ is the regression coefficient of explanatory variables $x_{j}$.

The incidence rate ratio (IRR) with its 95% confidence interval (CI) for per 0.1 unit increment in greenness was reported. All the covariates were scaled. GDP per capita and population density were also log-transformed due to severe skewness. The Spearman correlation coefficient (r_s_) and variance inflation factor (VIF) were calculated to diagnose the potential multicollinearity among multiple variables (Table S2 and S3). The main model was performed by removing highly correlated variables (r_s_ ≥ 0.6). We used a threshold value of 5 for VIF to identify the presence of multicollinearity.

Stratified analyses by population density, GDP per capita, and urbanization rate were performed to investigate the effect modification. We categorized these covariates into two groups based on the median. The effect modification was tested by adding interaction as a product term into the model, which was adjusted for all the covariates, except for the interaction covariates. Mediation analysis was conducted to investigate the potential mediators (air pollutants) on the role of NDVI and COVID-19 incidence. The significance of the mediation effect was tested through 500 bootstraps resamples of the estimated indirect effect. We reported the average direct effect (ADE) and average causal mediation effect (ACME).

To test the robustness of our results, we performed a series of sensitivity analyses. First, to assess the sensitivity of the main model to confounders, we removed the confounders in turn. Second, we excluded several cities with less than 10 and 5 confirmed COVID-19 cases. Third, NDVI in the winter season (January and February) might not completely represent the level of greenness in area with snow cover. We also obtained the NDVI data from July to September 2019, the greenest months in one year. The association of NDVI in this period with COVID-19 incidence was also assessed.

All the statistical analyses were conducted in R statistical software, Version 4.0.3 (University of Auckland, New Zealand) using lme4 package (for main model analysis) or mediation package (for mediation analysis). Research ethics board approval was not required because all data were publicly available and aggregated at the city level.

## 3 Results

### 3.1 Descriptive statistics

**Fig. 1:**
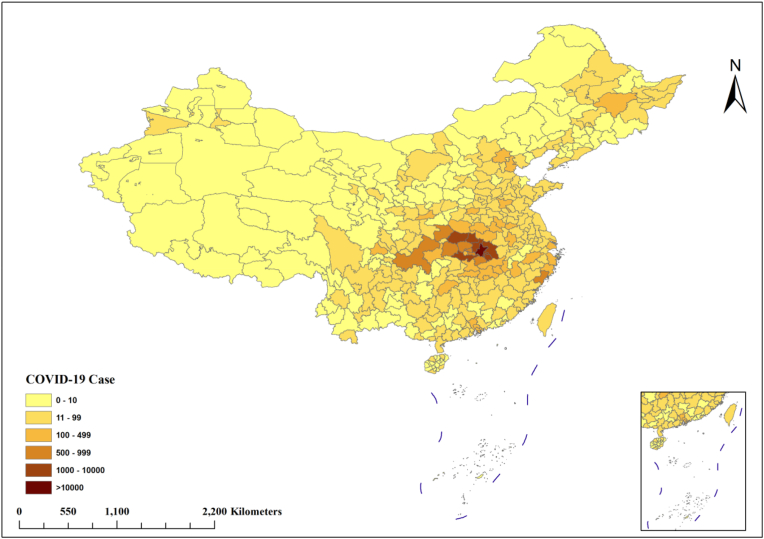
COVID-19 confirmed cases maps of China.

**Fig. 2:**
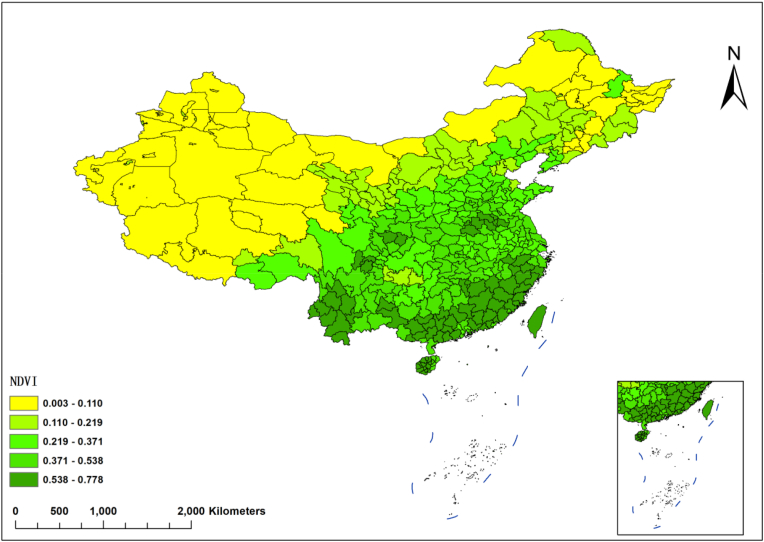
Spatial distribution of city-level NDVI in China.

A total of 266 cities were enrolled in our study. 12,377 confirmed COVID-19 cases were identified through February 29th, 2020. The COVID-19 incidence per 100,000 individuals was 0.98. Table 1 presented the descriptive statistics of the characteristics of 266 cities. 70 cities had 10 or fewer and 28 had 5 or fewer confirmed cases. The median value of NDVI during January and February 2020 was 0.387. Fig. 1 displayed the spatial distribution of COVID-19 confirmed cases in China. Except for Hubei province as the center of the outbreak, COVID-19 incident cases were more prevalent in municipalities (e.g. Chongqing, Beijing, and Shanghai) and the capital cities of provinces. Fig. 2 visualized the spatial variation of NDVI in China. Generally, greenness density was more prominent in the eastern and southern cities of the country.

**Table 1:** Descriptive statistics of 266 cities.

Variables	Median (P25, P75)
COVID-19 confirmed cases	12,377
COVID-19 incidence (per 100,000)	0.98
NDVI	0.387 (0.244, 0.521)
Population density (person/sq. km.)	335.89 (167.69, 636.67)
Older people (%)	14.06 (12.17, 16.03)
Gender ratio	103.45 (101.62, 106.38)
Education years (years)	9.57 (9.16, 10.07)
Urbanization rate (%)	58.26 (50.96, 68.60)
GDP per capita (CNY)	50307.5 (36369.0, 79149.0)
Hospital beds (per 1,000)	4.90 (4.15, 5.74)
Doctors (per 1,000)	2.68 (2.23, 3.09)
GRI	42.93 (40.86, 44.38)
Intra-city movement intensity	3.76 (3.44, 4.09)
PM_2.5_ (μg/m^3^)	49.00 (33.50,75.50)
NO_2_ (μg/m^3^)	25.00 (19.00, 32.50)
CO (mg/m^3^)	0.86 (0.75, 1.10)
Temperature (°C)	5.63 (0.25, 9.62)
Relative humidity (%)	72.48 (60.63, 79.40)
Cities with confirmed cases less than 10 (n)	70
Cities with confirmed cases less than 5 (n)	28

Abbreviation: P25, the 25th percentile; P75, the 75th percentile; sq. km., square kilometer; CNY, Chinese Yuan.

### 3.2 Association between greenness exposure and COVID-19 incidence

**Fig. 3:**
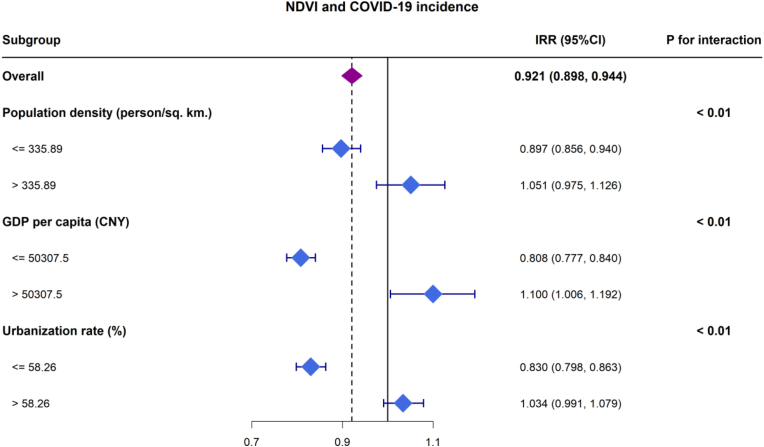
Stratified analyses on per 0.1 unit increment in NDVI and COVID-19 incidence. Except for the stratified covariates, all the stratified analyses were adjusted for population density, older people, GDP per capita, hospital beds, doctors, GRI, intra-city movement intensity, and temperature. Abbreviation: sq. km, square kilometer; CNY, Chinese Yuan.

Fig. 3 showed the results from the main analysis and stratified analysis. In the main adjusted model by population density, older people, GDP per capita, hospital beds, doctors, GRI, intra-city movement intensity, and temperature, we found an inverse association between city-level NDVI and COVID-19 incidence, with an IRR of 0.921 (95% CI: 0.898, 0.944) for per 0.1 unit increment in NDVI. Stronger associations were observed in cities with lower population density (IRR: 0.897, 95% CI: 0.856, 0.940), lower GDP per capita (IRR: 0.808, 95% CI: 0.777, 0.840) and lower urbanization rate (IRR: 0.830, 95% CI: 0.798, 0.863). All the interaction tests were significant.

### 3.3 Mediation analysis

Table 2 showed the mediation analysis by PM_2.5_, NO_2,_ and CO. The ADEs were statistically significant. We failed to detect any significant mediation effect of air pollutants on the association between NDVI and COVID-19 incidence.

**Table 2:** Mediation analysis of the association between NDVI and COVID-19 by air pollutants.

Mediator	ACME estimate (95% CI)	ADE estimate (95% CI)	Proportion mediated (95% CI) (%)	Proportion *P* value
PM_2.5_	−0.0003 (−0.0011,0.0005)	−0.0080 (−0.0124,-0.0046)	2.91 (−6.98,12.22)	0.52
NO_2_	0.0006 (−0.0016,0.0023)	−0.0114 (−0.0162,-0.0074)	−5.85 (−31.56, 11.13)	0.47
CO	−0.0003 (−0.0020,0.0014)	−0.0087 (−0.0130,-0.0052)	2.26 (−20.33,17.92)	0.72

Note: Adjustment for population density, older people, GDP per capita, hospital beds, doctors, GRI, intra-city movement intensity, and temperature. Abbreviation: ACME, average causal mediation effect; ADE, average direct effect.

### 3.4 Sensitivity analysis

The results from a series of sensitivity analyses were provided in Table S4. In the analysis omitting one covariate in turn, the results were consistent with the main analysis. When we excluded the cities with less than 10 and less than 5 confirmed cases, the results remained significant, with an IRR of 0.936 (95% CI: 0.912, 0.962) and 0.915 (95% CI: 0.891, 0.939), respectively. The significant association became more pronounced when we used NDVI from July to September 2019 as exposure measurement (IRR: 0.878, 95% CI: 0.842, 0.916).

## 4 Discussion

### 4.1 Main findings and interpretation

In this ecological study with 266 Chinese cities, we found that city-level greenness exposure was negatively associated with COVID-19 incidence. Per 0.1 unit increment of NDVI was associated with a 7.9% decrease in COVID-19 incidence. The results provided scientific evidence of the protective effect of greenness on infectious disease. Coincidentally, consistent with our results, studies from the United States and Canada also suggested a negative association of greenness with COVID-19 incidence (Ciupa and Suligowski, 2021; Klompmaker et al., 2021; Stieb et al., 2020). In addition, several studies found an inverse association between greenness and COVID-19 mortality (Klompmaker et al., 2021; Russette et al., 2021). Existing evidence was mainly conducted in developed countries. This is the first study to investigate the association between greenness exposure and COVID-19 incidence in the China region.

In stratified analyses, we found a stronger protective relation of NDVI on COVID-19 incidence in cities with lower population density. The result was not consistent with an ecological study based on approximately 3000 counties that found stronger associations in densely populated counties (Klompmaker et al., 2021), and they explained a positive association of greenness and COVID-19 mortality in the lowest population density that an increase in greenness limited the access to health care. Differences in the association might be due to the heterogeneity of greenness variation and other confounders. The population is relatively stable in cities with lower population density in China. Individuals living in these areas have fewer opportunities to contact the nonnative population. Studies (Frank and Engelke, 2005; Shen et al., 2017) suggested that the increase in population density had a positive relationship with the accumulation of air pollutants, which increased the risk of COVID-19 infection. When stratified by GDP per capita, we found a stronger association in cities with lower GDP per capita. The GDP per capita is often considered a reflection of regional living standards. Similar to previous studies (Maas et al., 2006; McEachan et al., 2016; Sarkar, 2017), health benefits of greenness were more prominent in populations with low income and low social-economic status. A possible explanation for this finding is that individuals with a lower socioeconomic status tend to be less mobile than those with higher counterparts, and spend more time in the vicinity of their homes, which promoted them contact with green space frequently (Maas et al., 2009).

### 4.2 Potential mechanism

Numerous studies have investigated the association of air pollutants with various COVID-19 outcomes in different regions. In general, exposure to the high concentration of air pollutants was associated with increased incidence (Adhikari and Yin, 2020; Vasquez-Apestegui et al., 2020; Zhu et al., 2020), mortality (Konstantinoudis et al., 2021; Liang et al., 2020; Wu et al., 2020) and case fatality (Liang et al., 2020; Tian et al., 2021; Yao et al., 2020). In contrast, exposure to more greenness has been suggested to reduce air pollutants (Dadvand et al., 2012; Nowak et al., 2006). One possible mechanism of greenness associated with decreased COVID-19 incidence was attributed to absorb air pollutants. Air pollution exposure could trigger oxidative stress and induce inflammation reactions, which may eventually deteriorate the immune system (Qin et al., 2020). Air pollution exposure has also been associated with increased infection of SARS-CoV-2 (the virus that causes COVID-19). In our mediation analysis, we failed to detect any significant mediation effects of air pollutants on NDVI and COVID-19 incidence association. Due to the unavailable of individuals’ residential addresses, the air pollutants were collected and averaged at the scale of city-level, which was inevitable to bring into potential misclassification bias. In addition, China adopted strict measures to control the spread of COVID-19 during the initial phase of COVID-19 outbreak. The closure of factories and the restriction of vehicles lead to the reduction of air pollutants. This might be one of the explanations for the non-significant mediation effect by air pollutants in our study. More mechanistic studies on greenness – air pollutants – COVID-19 association are therefore needed to address the confusion.

### 4.3 Strengths and limitations

There are two main strengths in our study. First, to our knowledge, this is the first study to quantitatively assess the association of greenness exposure with COVID-19 incidence in the China region. Second, we have performed a series of sensitivity analyses through the exclusion of cities with less than 10 and 5 confirmed COVID-19 cases and using greenness data from other months. The results remained robust and consistent.

However, several limitations should be acknowledged. First, this study is an ecological study with aggregated data at the city level. We could not obtain the individual-level confounding factors. Thus, the results might be affected by the ecological fallacy, which might bias the results to derive the causality. Second, we used NDVI as a proxy measurement of greenness, which could not provide information about the composition of green spaces, such as parks, forests, and agricultural land, and also the structure of vegetation, such as trees, grass, and shrubs. Third, epidemic control strategies across cities did not take into account, such as the closure of community, wearing masks, banning orders, and stay-at-home order. However, we think that there are few differences in the control strategies with the strict intervention measures implemented by the Chinese government. In addition, we obtained two indices, namely GRI and intra-city movement intensity, as a proxy of keeping social distance and population movement. Last but not the least, the first wave outbreak of COVID-19 was occurred during the period of the Chinese New Year, experiencing the largest population mobility. Assignment of greenness exposure was inevitable to bring potential misclassification bias. Thus, based on the above limitation, the results from our study should be interpreted with caution, and need to validate in other studies with less bias.

## 5 Conclusions

In conclusion, our study suggested a beneficial association between city-level greenness and COVID-19 incidence. This association was stronger in cities with lower population density, lower GDP per capita, and lower urbanization rate. Although causality was unable to be derived from this study, the results might be important toward increasing more greenness and decreasing air pollution to protect human health. It is important for public health implications to research how modifiable factors are associated with COVID-19 outcomes to guide policymaking. Further studies on the individual level that can access plausible biologic mechanisms are needed to uncover the protective effect of greenness.

## Credit author statement

**Wenjia Peng**: Conceptualization, Software, Formal analysis, Writing – original draft. **Yilin Dong**: Data curation, Software, Writing – original draft. **Meihui Tian**: Data curation, Writing – original draft. **Jiacan Yuan**: Resources, Writing – review & editing. **Haidong Kan**: Resources, Writing – review & editing. **Xianjie Jia**: Resources, Writing – review & editing. **Weibing Wang**: Conceptualization, Resources, Writing – review & editing, Project administration, Funding acquisition.

## Funding

This study was granted by 10.13039/100000865Bill & Melinda Gates Foundation, Seattle, WA, United States (Grant No. INV-006277), 10.13039/501100001809National Natural Science Foundation of China, China (Grant No. 82073612), and Shanghai New Three-year Action Plan for Public Health, China (Grant No. GWV-10.1-XK16).

## Data availability

The data that support the findings of this study are available from publicly available sources.

## Declaration of competing interest

The authors declare that they have no known competing financial interests or personal relationships that could have appeared to influence the work reported in this paper.
