# Supplementary material for: City-level greenness exposure is associated with COVID-19 incidence in China
Source: Environ Res. 2022 Jun;209:112871. doi: 10.1016/j.envres.2022.112871 (PMC8812109; doi:10.1016/j.envres.2022.112871)
Supplement: Multimedia component 1 [file mmc1.docx]

**Supplementary Materials for**

**City-level greenness exposure is associated with COVID-19 incidence in China**

Wenjia Peng, Yilin Dong, Meihui Tian, Jiacan Yuan, Haidong Kan, Xianjie Jia, Weibing Wang ^*^

^*^Corresponding author: Department of Epidemiology, School of Public Health, Fudan University (E-mail: [wwb@fudan.edu.cn](mailto:wwb@fudan.edu.cn))

**Contents**

Table S1 Descriptive and source of the outcome, exposure, potential covariates, air pollutants, and meteorological factors

Table S2 Spearman correlation coefficients among candidate covariates

Table S3 Results from a generalized linear mixed-effects model

Table S4 Sensitivity analysis results

Table S1 Descriptive and source of the outcome, exposure, potential covariates, air pollutants, and meteorological factors

| Variable | Descriptive | Source |
| --- | --- | --- |
| Outcome |  |  |
| COVID-19 cases | COVID-19 Confirmed cases by RT-PCR from the first case from January 1^st^ to February 29^th^, 2020 | National Health Commission and Provincial Health Commissions |
| Exposure |  |  |
| Greenness | City-level NDVI from January 1^st^ to February 29^th^, 2020 | MOD13A2 product at 1000-m resolution  <https://ladsweb.modaps.eosdis.nasa.gov/search/> |
| Potential covariates |  |  |
| Population density | Defined as total population divided by land area.  Unit: person/sq. km. | The seventh national census data from national, provincial, municipal statistical bureau |
| Older people | Proportion of older adults aged over 65 years old |  |
| Gender ratio | Ratio of Male : female (100) |  |
| Education years | Average education years of the population aged over 15 years old |  |
| Urbanization rate | Defined as the rate of urban population |  |
| GDP per capita | City-level gross domestic product per person  Unit: Chinese Yuan | Provincial, municipal statistical yearbook of 2019 |
| Hospital beds | Hospital beds per thousand population |  |
| Doctors | The number of licensed doctors per thousand population |  |
| GRI | Government response index | The Coronavirus Government Response Tracker |
| Intra-city movement  intensity | The proportion of people traveling within cities from January 1^st^ to February 29^th^, 2020 | Baidu map migration big data  http://qianxi.baidu.com/ |
| Air pollutants |  |  |
| PM_2.5_ | [Mean concentration](javascript:;) of particulate matter with an aerodynamic diameter less than or equal to 2.5 μm from January 1^st^ to February 29^th^, 2020 | National, provincial and municipal Bureau of Ecological Environment |
| CO | [Mean concentration](javascript:;) of carbon monoxide from January 1^st^ to February 29^th^, 2020 |  |
| NO_2_ | [Mean concentration](javascript:;) of nitrogen dioxide from January 1^st^ to February 29^th^, 2020 |  |
| Meteorological factors |  | China Meteorological Data Sharing Service System |
| Temperature | Mean temperature from January 1^st^ to February 29^th^, 2020 |  |
| Relative humidity | Mean Relative humidity from January 1^st^ to February 29^th^, 2020 |  |

Table S2 Spearman correlation coefficients among candidate covariates

| Variables | Population density | Older people | Gender ratio | Education years | Urbanization rate | GDP per capita | Hospital beds | Doctors | GRI | Intra-city  Movement  intensity | Temperature | Relative  humidity |
| --- | --- | --- | --- | --- | --- | --- | --- | --- | --- | --- | --- | --- |
| Population density | 1.00 | -0.13 | 0.10 | 0.29 | 0.37 | 0.52 | -0.25 | 0.03 | 0.11 | -0.29 | 0.32 | 0.25 |
| Older people | -0.13 | 1.00 | **-0.62** | -0.16 | -0.11 | -0.18 | 0.36 | 0.19 | 0.17 | 0.35 | -0.34 | -0.07 |
| Gender ratio | 0.10 | **-0.62** | 1.00 | 0.04 | 0.11 | 0.29 | -0.37 | -0.26 | -0.14 | -0.11 | 0.58 | 0.37 |
| Education years | 0.29 | -0.16 | 0.04 | 1.00 | **0.73** | 0.55 | 0.22 | **0.60** | -0.01 | -0.47 | -0.22 | -0.30 |
| Urbanization rate | 0.37 | -0.11 | 0.11 | **0.73** | 1.00 | **0.73** | 0.17 | 0.49 | 0.15 | -0.45 | -0.05 | -0.10 |
| GDP per capita | 0.52 | -0.18 | 0.29 | 0.55 | **0.73** | 1.00 | 0.02 | 0.32 | 0.11 | -0.29 | 0.25 | 0.12 |
| Hospital beds | -0.25 | 0.36 | -0.37 | 0.22 | 0.17 | 0.02 | 1.00 | 0.53 | -0.01 | 0.06 | -0.41 | -0.25 |
| Doctors | 0.03 | 0.19 | -0.26 | **0.60** | 0.49 | 0.32 | 0.53 | 1.00 | 0.11 | -0.25 | -0.44 | -0.40 |
| GRI | 0.11 | 0.17 | -0.14 | -0.01 | 0.15 | 0.11 | -0.01 | 0.11 | 1.00 | -0.12 | -0.24 | -0.14 |
| Intra-city  Movement  intensity | -0.29 | 0.35 | -0.11 | -0.47 | -0.45 | -0.29 | 0.06 | -0.25 | -0.12 | 1.00 | 0.03 | 0.14 |
| Temperature | 0.32 | -0.34 | 0.58 | -0.22 | -0.05 | 0.25 | -0.41 | -0.44 | -0.24 | 0.03 | 1.00 | **0.84** |
| Relative  humidity | 0.25 | -0.07 | 0.37 | -0.30 | -0.10 | 0.12 | -0.25 | -0.40 | -0.14 | 0.14 | **0.84** | 1.00 |

Table S3 Results from a generalized linear mixed-effects model

| Variables | Estimates | Std. Error | P value | IRR (95% CI) | VIF |
| --- | --- | --- | --- | --- | --- |
| NDVI | -0.083 | 0.013 | <0.001 | 0.921(0.898,0.944) | 1.53 |
| Population density | 0.028 | 0.025 | 0.247 | 1.029(0.980,1.079) | 2.81 |
| Older people | 0.041 | 0.021 | 0.049 | 1.042 (1.000,1.084) | 2.29 |
| GDP per capita | 0.022 | 0.018 | 0.224 | 1.022 (0.987,1.059) | 2.54 |
| Hospital beds | -0.162 | 0.018 | <0.001 | 0.850 (0.821,0.881) | 1.59 |
| Doctors | 0.084 | 0.016 | <0.001 | 1.087(1.055,1.121) | 2.45 |
| GRI | 0.155 | 0.083 | 0.061 | 1.168 (0.993,1.374) | 1.07 |
| intra-city movement intensity | -0.264 | 0.017 | <0.001 | 0.768 (0.743,0.795) | 2.09 |
| Temperature | 0.410 | 0.057 | 0.001 | 1.507 (1.347,1.686) | 1.23 |

Table S4 Sensitivity analysis results

| Analysis | IRR (95% CI) | P-value |  |
| --- | --- | --- | --- |
| Main analysis | 0.921 (0.898, 0.944) | <0.001 |  |
| Exclude Hospital beds | 0.945 (0.922, 0.969) | <0.001 |  |
| Exclude population density | 0.917 (0.895, 0.940) | <0.001 |  |
| Exclude GDP per capita | 0.917 (0.895, 0.940) | <0.001 |  |
| Exclude older people | 0.921 (0.898, 0.945) | <0.001 |  |
| Exclude GRI | 0.921 (0.898, 0.944) | <0.001 |  |
| Exclude doctors | 0.945 (0.923, 0.967) | <0.001 |  |
| Exclude intra-city movement intensity | 0.906 (0.884, 0.929) | <0.001 |  |
| Exclude temperature | 0.946 (0.923, 0.969) | <0.001 |  |
| Exclude cities with < 10 confirmed cases | 0.936 (0.912, 0.962) | <0.001 |  |
| Exclude cities with < 5 confirmed cases | 0.915 (0.891, 0.939) | <0.001 |  |
| NDVI from July to September 2019 | 0.878 (0.842, 0.916) | <0.001 |  |
